# Are children with developmental dyslexia all the same? A cluster analysis with more than 300 cases

**DOI:** 10.1002/dys.1629

**Published:** 2019-07-22

**Authors:** David Giofrè, Enrico Toffalini, Serena Provazza, Antonio Calcagnì, Gianmarco Altoè, Daniel J. Roberts

**Affiliations:** ^1^ Department of Educational Sciences University of Genoa Genoa Italy; ^2^ Department of General Psychology University of Padova Padova Italy; ^3^ Natural Sciences and Psychology Liverpool John Moores University Liverpool UK; ^4^ Department of Developmental and Social Psychology University of Padova Padova Italy; ^5^ Centre for Cognitive Neuroscience, Division of Psychology, College of Health and Life Sciences Brunel University London Uxbridge UK

**Keywords:** Developmental dyslexia (DD), specific learning disabilities (SLD), WISC‐IV, triangle model, dual‐route model (DRC)

## Abstract

Reading is vital to every aspect of modern life, exacerbated by reliance of the internet, email, and social media on the written medium. Developmental dyslexia (DD) characterizes a disorder in which the core deficit involves reading. Traditionally, DD is thought to be associated with a phonological impairment. However, recent evidence has begun to suggest that the reading impairment in some individuals is provoked by a visual processing deficit. In this paper, we present WISC‐IV data from more than 300 Italian children with a diagnosis of DD to investigate the manifestation of phonological and visual subtypes. Our results indicate the existence of two clusters of children with DD. In one cluster, the deficit was more pronounced in the phonological component, while both clusters were impaired in visual processing. These data indicate that DD may be an umbrella term that encompasses different profiles. From a theoretical perspective, our results demonstrate that dyslexia cannot be explained in terms of an isolated phonological deficit alone; visual impairment plays a crucial role. Moreover, general rather than specific accounts of DD are discussed.

## 1 INTRODUCTION

In order to lead a normal life in most societies, it is vital to have an appropriate level of reading skills. Developmental dyslexia (DD) affects 15% of individuals worldwide (American Psychiatric Association, 2013) and forms a major minority group. Although the central diagnostic phenotype (reading) is to some extent homogeneous and well established, the neurocognitive cause has been defined rather narrowly (Ramus, Altarelli, Jednorog, Zhao, & di Covella, 2018). The existence of distinct subgroups of DD has frequently been reported (Castles, Bates, & Coltheart, 2006; Castles & Coltheart, 1993; Coltheart & Kohnen, 2012; Friedmann & Coltheart, 2016; Kohnen et al., 2018) and there are two main varieties—those who suffer from a phonological deficit (lexically based) and those who suffer from a visual deficit. Most dyslexic individuals are classified under the lexically based subtype with the majority of studies reporting language‐related deficits in phonological processing.

**Figure 1:**
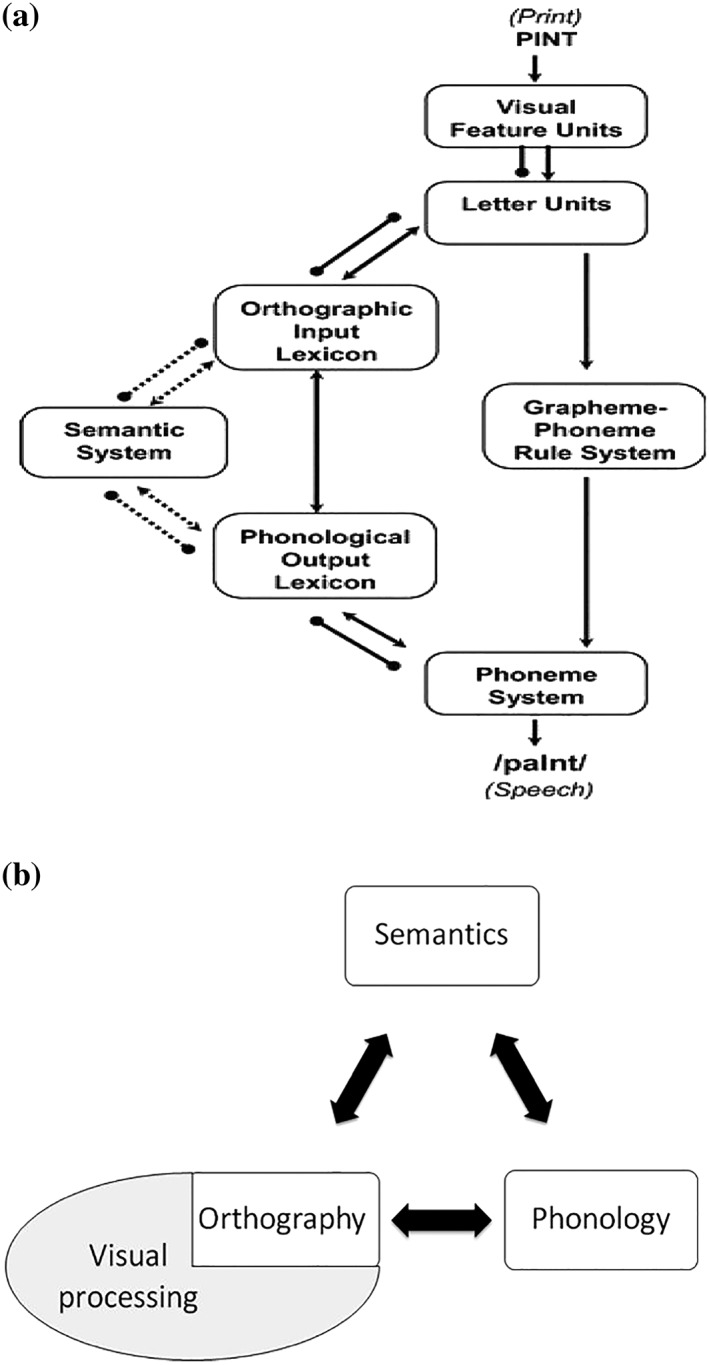
(a) DRC model (e.g., Coltheart and al., 2001) and (b) Triangle model (e.g., Seidenberg & McClelland, 1989; Plaut, McClelland, Seidenberg, & Patterson, 1996)

There is now considerable support for the idea that a phonological deficit is a major contributing factor to DD. The dual‐route reading framework (DRC; Coltheart, Rastle, Perry, Langdon, & Ziegler, 2001) has been highly influential in explaining the lexically based subtype (Figure 1a). In this model, reading can be achieved via two routes: (a) lexically through access to stored representations in the orthographic and phonological lexicons and (b) sub‐lexically through a phonological grapheme‐to‐phoneme conversion procedure. The lexical route permits reading of familiar words, whereas the sub‐lexical route processes unfamiliar words and phonologically plausible non‐words (e.g., *plur*) through a spelling‐to‐sound conversion mechanism. In this conceptualization, DD results from damage to specific components or stages of lexical processing—for example, to the sub‐lexical route or phonological lexicon, although damage to any component of the DRC may result in different reading difficulties. In keeping with the DRC framework, the majority of interventions are targeted at these components through speech and language therapy, which often leads to improvement in reading ability.

From a neural perspective, this fits with the long‐standing assumption that DD involves disruption of the left perisylvian phonological regions. Recent advances in neuroscience, however, have shown anatomical irregularities in the development, connectivity, or lateralisation of left occipitotemporal regions associated with visual processing (for review see: Ramus et al., 2018). Remarkably, visual processing *per se* is not routinely assessed in DD, but when it has been, impairments are apparent and in some instances without a phonological deficit (Behrmann & Plaut, 2015; Collins, Dundas, Gabay, Plaut, & Behrmann, 2017; Dundas, Gabay, Plaut, & Behrmann, 2014; Dundas, Plaut, & Behrmann, 2013; Gabay, Dundas, Plaut, & Behrmann, 2017). Moreover, the visual deficit is not restricted to words and extends to other visual stimuli (for a recent theoretical proposal see: Behrmann & Plaut, 2013; Plaut & Behrmann, 2011). Only a minority of DD individuals fall into this subtype but cases may have gone unnoticed due to insensitive tests, unrecognized diagnosis, data outliers, publication bias, and small sample sizes. A related issue concerns the cognitive model used to diagnose and remediate dyslexic individuals. Although distinctions between lexically based dyslexias can be explained and treated using the DRC approach, it cannot fully accommodate the visual subtype because the model is reading specific and does not incorporate non‐orthographic visual processing. Proponents of the DRC, however, would argue that if there is a problem in visual processing that affects all visual tasks, it is not, by definition, a reading problem. Yet, many cases of reading impairment that co‐occur with frank visual processing impairments have indeed been explained in a DRC framework in the past and designated several labels, including alexia without agraphia, agnostic alexia, word form dyslexia, verbal alexia, global alexia, word blindness, letter‐by‐letter reading/dyslexia, and spelling dyslexia (for review see Starrfelt & Shallice, 2014; Yong, Warren, Warrington, & Crutch, 2013). This also includes disruption purported to occur before the reading system.

By contrast, the primary systems view (Hoffman, Lambon Ralph, & Woollams, 2015; Patterson & Lambon Ralph, 1999) can accommodate both DD subtypes. This differs from the DRC in that reading is underpinned by the phylogenetically more mature primary systems of vision, phonology, and semantics. The triangle model (Figure 1b) is an instantiation of this approach, implemented in a parallel distributed processing (PDP) connectionist network (Plaut et al., 1996). Central to this approach is the proposal that the same computational elements, in various combinations, support different activities: (a) vision, which with respect to reading mediates knowledge about orthographic word form, (b) phonology—the internal representation of word sound, and (c) semantics—word meaning. Reading aloud can be accomplished directly between vision and phonology (V>P) or mediated by semantics (V>S or the interplay between S<>P). During reading acquisition, the direct pathway becomes sensitive to the relationship that exists between graphemes and phonemes and achieves efficient computations for regular words and non‐words with typical grapheme–phoneme rules (e.g., *pat* and *snat*). It is less efficient for infrequent irregular words with atypical grapheme–phoneme rules (e.g., *poignant*) and it is these that require additional semantic support.

The application of the cognitive approach in explaining DD has proved valuable with reports of word length effects and visual impairments in DD (Dehaene, Cohen, Morais, & Kolinsky, 2015; Gabay, Dundas, Plaut, & Behrmann, 2017; Provazza, Adams, Giofre, & Roberts, 2019; Provazza, Giofre, Adams, & Roberts, 2019). Furthermore, similar brain abnormalities (e.g., left vOT) have been noted across multiple methods including total brain volume, voxel‐ and surface‐based morphometry, white matter, diffusion imaging, brain gyrification, and tissue metabolite (for review, see Ramus et al., 2018). Consequently, an association seems to exist between the neural bases of dyslexia and visual and phonological impairments.

Deficits in verbal memory tasks have been frequently found in children with DD (Kipp & Mohr, 2008; Vellutino, 2004), and this finding is corroborated by neuroimaging studies (e.g., Beneventi, Tønnessen, Ersland, & Hugdahl, 2010). Key processes in learning to read involve coding, storage, and retrieval of stable associations between speech and written language (Elliott & Grigorenko, 2014). Several studies have indicated that phonological memory skills and speech and language skills are highly correlated, and the increase in verbal memory skills is linked to an increase in speech and language skills (Grivol & Hage, 2011; Hulme, Thomson, Muir, & Lawrence, 1984). There is also research indicating that digit‐span tasks, such as those included in the WISC‐IV, require a lower level of cognitive control as compared with dual tasks test (e.g., Engle, Tuholski, Laughlin, & Conway, 1999). This finding corroborates the idea that the digit span task requires verbal abilities and—to a latter extent—cognitive control resources (see Giofrè, Mammarella, & Cornoldi, 2013; and Cornoldi & Giofrè, 2014 for a review).

Crucially important for lexical retrieval implied in reading processes is visual recognition of letters and symbols. Deficits in children with DD seem not to be limited to phonological abilities but encompass a variety of other subtle deficits, for example, with visuospatial materials (Menghini, Finzi, Carlesimo, & Vicari, 2011). It has been shown that children with DD struggle in tasks, such as coding, which involve visual abstract symbols (e.g., Piazza et al., 2010; Gubbay & de Klerk, 1995; Valdois, Bosse, & Tainturier, 2004). A similar argument can be found for tasks, such as symbol search, in which the presence of subtle visual search deficits has often been associated with reading problems (e.g., Casco, Tressoldi, & Dellantonio, 1998). Both coding and symbol search, are included in the so‐called processing speed index in the WISC‐IV and seem to also tap executive resources. However, as compared with other tasks included in the WISC‐IV, the cognitive control required in these tasks seem to be lower; that is, these tasks seem to have lower loadings on the g‐factor (Giofrè & Cornoldi, 2015). Hence, it is conceivable to consider tasks included in the WISC‐IV as particularly suitable for testing the presence of potential phonological and visual deficits, as currently underpinned by the large number of studies using these materials in children with DD.

It is currently unclear how these impairments contribute to the different subtypes of DD, partly because children are classified under the same umbrella term (American Psychiatric Association, 2013; Elliot & Gibbs, 2008). Instead of phonological (lexically based) and visual subtypes, we propose a continuum of phonological–visual impairment. It should be noted, however, that in this context, the phonological subtype includes all DD who present with a deficit in verbal abilities, not only those described as phonological dyslexics in the DRC model. Depending on the neuroanatomical bases (D'Souza & Karmiloff‐Smith, 2017; Ramus et al., 2018), the point one falls along the continuum may determine the unique features present in the dyslexic profile. Recent evidence from children with specific learning disabilities shows that different cognitive clusters are apparent in this group (Poletti, Carretta, Bonvicini, & Giorgi‐Rossi, 2018). However, to the best of our knowledge, a cluster analysis on children with a specific diagnosis of DD employing the Wechsler Intelligence Scale for Children (WISC‐IV) has not been performed on phonological–visual factors.

The possibility to identify the presence of phonological–visual subgroups or mixed phonological–visual subgroups therefore needs further clarification and is dependent on recruiting a sufficiently large sample of DD individuals. To address this issue, we collated data from 316 children on the WISC‐IV (Wechsler, 2003), which contains visual and phonological subtests. The main aim of the study, therefore, is to evaluate the presence of different clusters of children with a diagnosis of DD.

## 2 METHOD

### 2.1 Participants

The WISC‐IV intellectual profiles of 316 children diagnosed with specific reading disorder (*M*_age_ = 11.72 years, *SD* = 2.61 years; age range 7–16 years; 55% males) were analysed. Only children who had received a diagnosis corresponding to the F81.0 category (also known as reading disorder or DD) of the ICD‐10 coding system (World Health Organization, 1992) were considered. Any case presenting comorbid neuropsychological condition coded by the ICD‐10 (including attention‐deficit/hyperactivity disorder, coordination disorder, specific language impairment) was excluded from the sample. The data were selected from a larger dataset including the WISC‐IV profiles of 1,414 children with different subtypes of learning disorder. Subsets of the entire dataset were analysed in previously published articles (Cornoldi, Orsini, Cianci, Giofrè, & Pezzuti, 2013; Giofrè & Cornoldi, 2015; Giofrè, Stoppa, Ferioli, Pezzuti, & Cornoldi, 2016; Giofrè, Toffalini, Altoè, & Cornoldi, 2017; Toffalini, Giofrè, & Cornoldi, 2017a; Toffalini, Pezzuti, & Cornoldi, 2017), but none of the analyses discussed in this paper were the subject of said previous reports. All data were provided by licensed psychologists with expertise in learning disabilities, working in clinical centres located in eight major Italian regions. In line with the National Italian Consensus Conference on Specific Learning Disorder by the Italian Ministry of Health (Istituto Superiore di Sanità, 2011), all children diagnosed with specific reading disorder were reported as (a) having academic achievement in reading below the 5th percentile in accuracy and/or two *SD*s below average in speed, as assessed using standardized tests appropriate for age and (b) not presenting major influence of known sociocultural, educational, emotional, intellectual, and neurological problem.

### 2.2 Instrument

The Italian standardization of the WISC‐IV (Orsini, Pezzuti, & Picone, 2012) was used. We examined the scores obtained in the 10 core subtests of the WISC‐IV—block design (BD), similarities (SI), digit span (DS), picture concepts (PCn), coding (CD), vocabulary (VC), letter–number sequencing (LN), matrix reasoning (MR), comprehension (CO), and symbol search (SS). We calculated the Full‐Scale IQ (FSIQ) from the sum of the 10 subtests, and the four‐factor indexes: the Perceptual Reasoning Index (PRI), which includes BD, PCn, and MR; the Verbal Comprehension Index (VCI), including SI, VC, and CO; the Working Memory Index (WMI) including DS and LN; and the Processing Speed Index (PSI) including CD and SS. In particular, the four main indices (VCI, PRI, WMI, and PSI), were calculated and were considered for the purposes of the cluster analysis. Of theoretical importance here is working memory (WMI), which is tapping phonology (Spring, 1976; Vargo, Grosser, & Spafford, 1995) and processing speed, which requires a fast response from the visual system (PSI).

## 3 RESULTS

To examine the underlying structure of VCI, PRI, WMI, and PSI, we used a model‐based clustering analysis approach, where clusters are modelled as a finite mixture of Gaussian distributions (Fraley & Raftery, 1998). Unlike standard clustering approaches (e.g., Ward), this analysis allows for a better partitioning of the data while retaining as much as possible from the data variability. This is performed by modelling data partitions by means of suitable covariance structure shapes (e.g., spherical and ellipsoid). The analysis was performed using the mclust package in R environment (Scrucca, Fop, Murphy, & Raftery, 2016), which allowed to evaluate different clustering solutions in terms of model parameters. To further corroborate the findings, additional methods (i.e., standard hierarchical cluster with Ward's method) were also used, which yielded the same results with regards to the number of clusters. These further results are not reported here, as they are redundant. Several clustering models (EII: spherical, equal volume; EEI: diagonal, equal volume and shape; VII: spherical, unequal volume; VEI: diagonal, varying volume, equal shape) were evaluated in terms of their evidence (as indicated by the BIC index, which quantifies the plausibility of a model given the data over a set of possible models) and number of underlying components (i.e., number of clusters): 1, 2, and 3. In the package mclust, the BIC index is computed with an alternative formulation so that the best model is the one with the highest score (e.g., in case of two models with scores −80 and −10, then the highest score associated to the best model is −10). Table 1 shows results for the models selection. The EII model with diagonal, equal volume and shape covariance matrix was selected as suggested by the BIC values. It identifies two components (i.e., two clusters). Table 2 shows the descriptive statistics for the subtests and the four indices VCI, PRI, WMI, and PSI of the children categorized in each of these two clusters.

**Table 1:** BIC for clustering models as function of the number of components

Number of Components	Models			
	EII	EEI	VII	VEI
1	−10,147.07	−10,157.47	−10,147.07	−10,157.47
2	−10,069.87	−10,073.46	−10,073.34	−10,077.00
3	−10,088.59	−10,094.27	−10,091.54	−10,097.04

*Note.* The models differ in terms of covariance matrix specification. EII: spherical, equal volume. EEI: diagonal, equal volume and shape. VII: spherical, unequal volume. VEI: diagonal, varying volume, equal shape. Model parameters were estimated via Expectation‐Maximization. For further information, see Scrucca et al. (2016).

**Table 2:** Descriptive statistics of the children with dyslexia categorized into two clusters

Measure	Cluster 1 (N = 113, 44% females)		Cluster 2 (N = 203, 45% females)	
	M	SD	M	SD
Age (months)	135.62	28.51	143.43	32.46
Subtests				
Similarities	12.47	2.57	9.21	2.26
Vocabulary	12.53	2.01	9.42	2.29
Comprehension	13.11	2.68	9.99	2.72
Block design	13.02	2.30	10.28	2.38
Picture concepts	13.04	2.36	10.46	2.62
Matrix reasoning	13.42	2.45	10.23	2.58
Digit span	9.86	2.53	7.71	2.18
Letter–number ordering	10.35	2.35	8.46	1.90
Coding	9.02	2.86	8.72	2.61
Symbol search	9.92	2.75	9.41	2.38
Indices				
Verbal comprehension	116.59	11.45	97.04	10.55
Perceptual reasoning	120.24	9.70	101.67	10.03
Working memory	101.03	11.61	88.18	10.61
Processing speed	96.46	13.63	94.44	12.20
Full scale IQ	113.56	7.07	94.77	7.23

*Note.* Minor differences in the index mean scores vis‐à‐vis Figure 2 are due to the fact that this table reports observed descriptive statistics, whereas Figure 2 shows model estimates.

**Figure 2:**
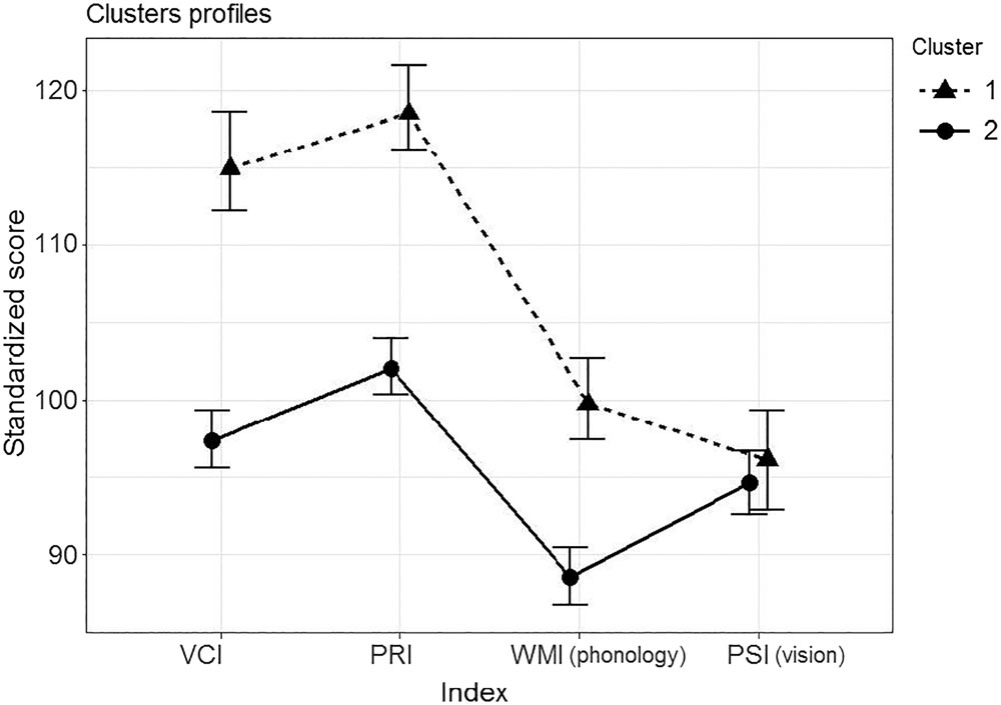
Mean scores and standard errors estimated by the model for the indices Verbal Comprehension Index, Perceptual Reasoning Index, Working Memory Index (phonology), and Processing Speed Index (vision) on the two clusters. Cluster 1, N = 113 and Cluster 2, N = 203. Error bars represent 95% confidence intervals of the estimated mean values, computed via non‐parametric bootstrap (with 5,000 samples generation)

Between‐group comparisons at the univariate level revealed that the two groups differed significantly in the IQ, *t*(314) = 22.31, *p* < .001, Cohen's *d* = 2.62, with Cluster 1 (*M* = 113.56, *SD* = 7.07) having a higher IQ compared with Cluster 2 (*M* = 94.77, *SD* = 7.23). To further investigate the intelligence profiles of the two clusters, we grouped participants on the basis of the individual classifications obtained from the selected clustering solution, and we conducted a one‐way (Group: Cluster 1 vs. Cluster 2) multivariate analysis of variance on VCI, PRI, WMI, and PSI as the dependent variables (see also Figure 2). As expected, a statistically significant difference emerged between the two groups at the multivariate level, Pillai's trace = 0.66, *F*(4, 311) = 153.12, *p* < .001. Between‐group comparisons at the univariate level revealed that the two groups differed significantly and with a large effect size in VCI, *t*(314) = 15.32, *p* < .001, Cohen's *d* = 1.80; PRI, *t*(314) = 15.96, *p* < .001, Cohen's *d* = 1.87; and WMI, *t*(314) = 9.97, *p* < .001, Cohen's *d* = 1.17, but not in PSI, *t*(314) = 1.35, *p* = .18, Cohen's *d* = 0.16.

### 3.1 Additional analyses

A cluster analysis on the larger dataset including children with other specific learning disabilities, but excluding children with DD has been performed (*n* = 1,098). This analysis showed the presence of only one cluster.

## 4 DISCUSSION

The aim of this study was to evaluate the presence of different clusters of children with DD. Additionally, to clarify whether the profile of children belonging to different clusters can be accounted for in terms of impairment to phonology and vision.

Our results indicate that children with DD can be disentangled into two main clusters. The first is characterized by children with higher IQs, with a PRI generally higher compared with the VCI, consistent with previous observations (Toffalini, Giofrè, & Cornoldi, 2017b). This cluster is also characterized by a striking dissociation of performance—a visual processing weakness coupled with intact phonological processing. The second cluster is characterized by lower IQs and shows the frank opposite severity pattern. Although weaknesses are apparent on both visual and phonological processing, more severe impairments are observed in the latter. These results seem to be robust and specific to children with DD. In fact, when the larger group of children with specific learning disabilities was considered, with the exclusion of children with DD, only one cluster emerged.

The existence of two clusters has important theoretical relevance. Although the two clusters present with polar patterns of impairment severity (Cluster 1: vision<phonology and Cluster 2: phonology<vision), which to all intents and purposes could be considered a double dissociation, they share a common feature—impaired visual processing. This indicates that children with DD may present with a general impairment in visual processing if tested appropriately—with some demonstrating the non‐phonological subtype. This is supported by previous studies which report a visual impairment in children with DD (e.g., Bosse, Tainturier, & Valdois, 2007; Stein & Walsh, 1997). We also frame our results within the triangle model, which postulates a co‐occurrence of deficit in more general underlying capacities in reading disorders as demonstrated in studies of patients with acquired dyslexia.

For instance, pure alexia is a selective disorder of reading and occurs as a consequence of damage to the left occipitotemporal cortex—the so‐called visual word form area (VWFA; Dehaene & Cohen, 2011). Several studies have disputed the selectivity of this disorder by demonstrating that individuals with pure alexia are also impaired in recognizing other visual stimuli (Behrmann, Nelson, & Sekuler, 1998; Roberts et al., 2013; Roberts et al., 2015), which also elicit activation in the VWFA in neurologically intact participants (for review and a computational implementation, see Behrmann & Plaut, 2013, 2014, 2015; Price & Devlin, 2011). Interestingly, individuals with DD have also been reported to show a hypo‐activation of the VWFA and an impairment in processing non‐verbal visual stimuli (e.g., Sigurdardottir et al., 2017) as, for instance, faces (e.g., Gabay et al., 2017). The measure of processing speed in WISC‐IV uses non‐verbal visual stimuli and the difficulties shown by children with DD on this task confirm a domain general impairment, supporting the predications of the triangle model (Woollams, 2014). Notably, children in the second cluster present with a dual‐system deficit in visual and phonological processing.

These results also have practical and clinical implications. In fact, the evaluation of the cognitive profile of children with DD can be beneficial for both the assessment and intervention of these children. As for the assessment, it can be of interest to compare the profile of a specific child with DD with clusters described in this paper. As for the intervention, previous attempts conducted using former versions of the WISC battery led to unsatisfactory results (e.g., Kavale & Forness, 1984; Watkins, Kush, & Glutting, 1997; cf. Koriakin et al., 2013; Lanfranchi, 2013). Therefore, large‐scale studies, where it is possible to individuate different clusters of children with DD, are needed to clarify this rehabilitation issue further.

This study is the first to demonstrate the existence of two distinct DD clusters, and we propose that the triangle framework provides the most optimal fit for our findings. The model also predicts that different orthographies rely differentially on vision, phonology, and semantics to support reading (e.g., Ziegler & Goswami, 2005), and we hypothesize that different manifestations of DD will be observed as a function of orthographic type. For instance, children learning Italian, a transparent orthography (graphemes directly map onto phonemes, e.g., *pinta* in which the phoneme/ɪ/is pronounced consistently like/pɪnta/) rely on visual processing and the direct triangle pathway (V>P). Conversely, children learning opaque and inconsistent orthographies such as English (not always a one‐to‐one mapping between graphemes–phonemes, e.g., *pint* could legitimately be pronounced/paɪnt/rather than/hɪnt/or/mɪnt/) are less reliant on vision but more on phonology and semantics, the indirect triangle pathway (V>S>P; S<>P; e.g., Helland & Morken, 2016; Marinelli, Romani, Burani, McGowan, & Zoccolotti, 2016). One indication of this comes from a study of 60 French children with DD. On the WISC‐IV, phonological impairments were more apparent than visual impairments (De Clercq‐Quaegebeur et al., 2010). This is entirely consistent with our prediction that opaque and transparent orthographies are reliant on phonological and visual processing differentially. It would also be of interest to evaluate the WISC‐IV profile of children with DD in other languages including English.

It is worth noting that the number of children belonging to the second cluster is higher compared with the number of children in the first cluster. This finding might have important implications. For example, some evidence indicates the presence of both verbal and visual deficits in poor readers (Menghini, Carlesimo, Marotta, Finzi, & Vicari, 2010; Swanson, 1999). However, findings tend to be inconsistent across different studies (e.g., Bell, 1990; Elliott & Grigorenko, 2014; Watson & Willows, 1995). One possible explanation for such discrepancies is that studies in this area tend to include only a limited number of participants. In fact, it might be argued that research with small sample sizes, as compared with the large number of participants included in the present report, might fail to identify visual deficits in children with DD, and this may explain the presence of inconsistent findings in the current literature. For this reason, future studies should endeavour to address this issue, for example, by including a large number of children with DD tested on several different tasks tapping both visual and phonological components.

Despite the considerable number of insightful findings, some limitations in the current report should be noted and addressed in future studies. First, this study did not include measures of external validity; it would be important to support these findings, including some other external measures to verify whether the two clusters that we identify diverge on a series of other tasks measuring more broadly visual and phonological components. Second, it would be important to include a variety of tasks manipulating, for example, the level of cognitive control required. In fact, tasks included in the WISC‐IV also tap other “executive” resources and it would be important to disentangle the relative impairment of children with DD on pure visual and phonological aspects by limiting the potential confounding of these executive resources.

Nonetheless, our study has confirmed the existence of different clusters of DD, as shown by previous literature (e.g., Heim et al., 2008). Moreover, differently from previous studies, the profile of a wider sample of children with DD has been analysed. This represents a novelty in the study of DD, since we employed a cognitive battery to identify phonological and visual deficits in DD. Finally, our findings strengthen the evidence that the diagnosis of DD is an umbrella term encompassing different subgroups. Consideration of these groups and profiles could potentially transform clinical diagnosis and treatment intervention approaches for DD. It may also extend to parallel reading impairments observed in different neurological groups such as those with acquired brain injury or dementia, both of which have been captured successfully in the neurocognitive triangular framework, albeit, due to different aetiological damage to the primary systems.
